# Supplementary material for: A network dynamical simulation model for the study of antibiotic resistance in microbial communities
Source: Gut Microbes. 2026 Sep 19;18(1):2734703. doi: 10.1080/19490976.2026.2734703 (PMC13596919; doi:10.1080/19490976.2026.2734703)
Supplement: Supplementary file 1 [file KGMI_A_2734703_SM9053.zip › supplementary_file_1/Figures/er_delta_mean_distance_gc_n100.pdf]

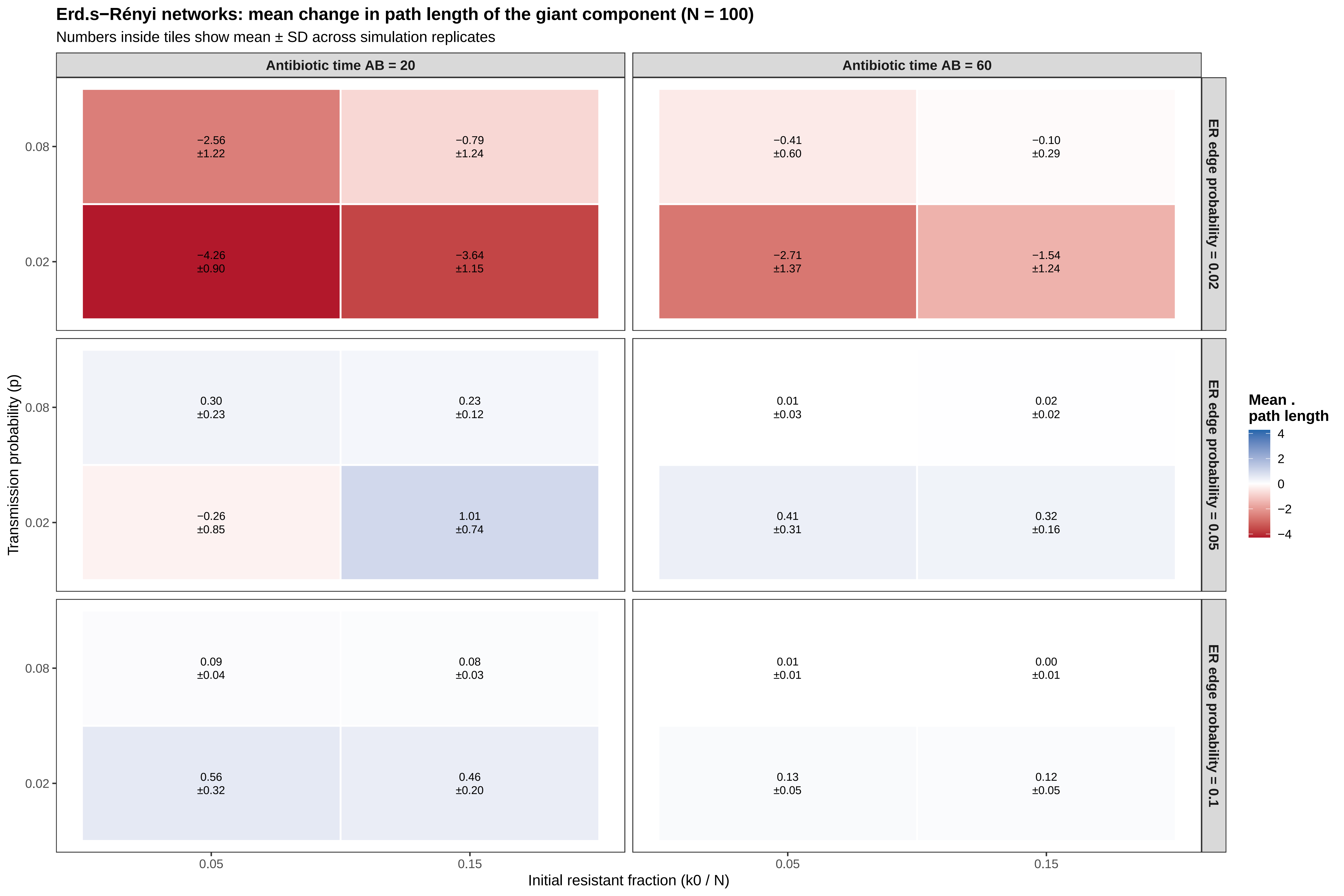
Erd.s−Rényi networks: mean change in path length of the giant component (N = 100)
Numbers inside tiles show mean ± SD across simulation replicates
Antibiotic time AB = 20
Antibiotic time AB = 60
ER edge probability = 0.02
ER edge probability = 0.05
ER edge probability = 0.1
−2.56 ±1.22
−0.79 ±1.24
−4.26 ±0.90
−3.64 ±1.15
−0.41 ±0.60
−0.10 ±0.29
−2.71 ±1.37
−1.54 ±1.24
0.30 ±0.23
0.23 ±0.12
−0.26 ±0.85
1.01 ±0.74
0.01 ±0.03
0.02 ±0.02
0.41 ±0.31
0.32 ±0.16
0.09 ±0.04
0.08 ±0.03
0.56 ±0.32
0.46 ±0.20
0.01 ±0.01
0.00 ±0.01
0.13 ±0.05
0.12 ±0.05
Transmission probability (p)
0.08
0.02
Initial resistant fraction (k0 / N)
0.05
0.15
Mean .
path length
4
2
0
−2
−4
